# Osteoblasts Promote Prostate Cancer Cell Proliferation Through Androgen Receptor Independent Mechanisms

**DOI:** 10.3389/fonc.2021.789885

**Published:** 2021-12-13

**Authors:** Giulia Ribelli, Sonia Simonetti, Michele Iuliani, Elisabetta Rossi, Bruno Vincenzi, Giuseppe Tonini, Francesco Pantano, Daniele Santini

**Affiliations:** ^1^ Department of Medical Oncology, Campus Bio-Medico University of Rome, Rome, Italy; ^2^ Department of Immunology and Molecular Oncology, Istituto Oncologico Veneto (IOV) Istituto di Ricovero e Cura a Carattere Scientifico (IRCCS), Padua, Italy; ^3^ Department of Surgery, Oncology and Gastroenterology, University of Padua, Padua, Italy

**Keywords:** castration resistance prostate cancer, bone microenvironment, androgen receptor, osteoblasts, matrix metalloproteinase-1

## Abstract

Patients with metastatic prostate cancer frequently develop bone metastases that elicit significant skeletal morbidity and increased mortality. The high tropism of prostate cancer cells for bone and their tendency to induce the osteoblastic-like phenotype are a result of a complex interplay between tumor cells and osteoblasts. Although the role of osteoblasts in supporting prostate cancer cell proliferation has been reported by previous studies, their precise contribution in tumor growth remains to be fully elucidated. Here, we tried to dissect the molecular signaling underlining the interactions between castration-resistant prostate cancer (CRPC) cells and osteoblasts using *in vitro* co-culture models. Transcriptomic analysis showed that osteoblast-conditioned media (OCM) induced the overexpression of genes related to cell cycle in the CRPC cell line C4-2B but, surprisingly, reduced androgen receptor (AR) transcript levels. In-depth analysis of AR expression in C4-2B cells after OCM treatment showed an AR reduction at the mRNA (*p* = 0.0047), protein (*p* = 0.0247), and functional level (*p* = 0.0029) and, concomitantly, an increase of C4-2B cells in S-G2-M cell cycle phases (*p* = 0.0185). An extensive proteomic analysis revealed in OCM the presence of some molecules that reduced AR activation, and among these, Matrix metalloproteinase-1 (MMP-1) was the only one able to block AR function (0.1 ng/ml *p* = 0.006; 1 ng/ml *p* = 0.002; 10 ng/ml *p* = 0.0001) and, at the same time, enhance CRPC proliferation (1 ng/ml *p* = 0.009; 10 ng/ml *p* = 0.033). Although the increase of C4-2B cell growth induced by MMP-1 did not reach the proliferation levels observed after OCM treatment, the addition of Vorapaxar, an MMP-1 receptor inhibitor (Protease-activated receptor-1, PAR-1), significantly reduced C4-2B cell cycle (0.1 μM *p* = 0.014; 1 μM *p* = 0.0087). Overall, our results provide a novel AR-independent mechanism of CRPC proliferation and suggest that MMP-1/PAR-1 could be one of the potential pathways involved in this process.

## Introduction

Androgen-deprivation therapy is the mainstay for advanced prostate cancer, but despite the initial success of these treatments, castration-resistant prostate cancer (CRPC) inevitably occurs within a few years (1). Multiple mechanisms of resistance contribute to the progression to castration-resistant disease and the androgen receptor (AR) remains the most important driver in this progression (2). At present, the approved chemotherapies for CRPC include systemic drugs (docetaxel and cabazitaxel) and agents that target androgen signaling such as enzalutamide, abiraterone, apalutamide, and daralutamide (3). Although these treatments confer a significant survival benefit, over time, the majority of patients inevitably develop resistance to treatment and their disease progresses. Several mechanisms have been attributed to these resistances including AR overexpression or mutations, the expression of constitutively active AR splice variants, the increase in intratumoral hormonal synthesis, and the activation of different growth factor pathways (4–7). At this stage, about 70% of patients develop bone metastases (8) usually associated with skeletal-related events (SREs) including pathological fractures, bone pain, and spinal cord compression that severely affect the patients’ quality of life (9). As widely described in vicious cycle theory, bone microenvironment represents a fertile soil where the bi-directional interaction between bone and cancer cells promotes tumor growth and progression (10–14). Bone metastases from prostate cancer are predominantly characterized by an increased osteoblast (OB) activation that, in turn, influences prostate cancer proliferation. Despite increasing evidence supporting a key role of OBs within bone metastatic niche (15–17), their precise contribution in supporting tumor cell survival and proliferation is not completely elucidated. From these perspectives, our purpose was to better elucidate the biohumoral interactions between OBs and prostate cancer cells in the bone microenvironment.

## Materials and Methods

### Prostate Cancer Cell Line

The C4-2B cell line was gently gifted by Thalman who isolated them in 1994 (18). C4-2B cells were grown in T-medium (80% Dulbecco’s modified Eagle’s medium, 20% F12K, 3 g/L NaHCO_3_, 100 units/L Penicillin G, 100 μg/ml Streptomycin, 5 μg/ml insulin, 13.6 pg/ml triiodothyronine, 5 μg/ml apo-transferrin, 0.25 μg/ml biotin, and 25 μg/ml adenine) with 10% FBS. The cells were Mycoplasma free. Green Fluorescent Protein C4-2B (C4-2B GFP+) cells were obtained transfecting cells with MISSION^®^ pLKO.1-puro-CMV Turbo GFP™ Positive Control Transduction Particles (multiplicity of infection: 0.5) (Sigma Aldrich). Transfected cells were isolated adding 2 μg/ml of Puromycin. C4-2B Firefly/Renilla (C4-2B FR) cells were generated transfecting C4-2B cells with androgen receptor (AR) two luciferase lentiviral particles using Cignal AR luciferase reporter assay (Qiagen). C4-2B FR were selected adding 100 μg/ml of Hygromycin and 2 μg/ml of Puromycin.

### Primary Human Osteoblasts

Human primary OBs were obtained from bone marrow samples of healthy patients undergoing total hip replacement at Policlinico Universitario Campus Bio-Medico of Rome, Italy. The procedure was approved by the Ethical Committee of the Campus Bio-Medico University of Rome and informed consent from patients was collected in accordance with the Declaration of Helsinki principles (Prot 21/15 OSS). Bone marrow mesenchymal stem cells (BM-MSCs) were isolated and differentiated in OBs as previously described (19). At the end of the OB differentiation protocol, the positivity for alkaline phosphatase (ALP) and Alizarin red staining were tested as previously described (20).

### OBs-C4-2B Cell Co-Cultures

For “indirect co-cultures”, osteoblast-conditioned media (OCM) and C4-2B conditioned-media (C4-2B CM) were collected respectively from OBs and C4-2B cells after 48 h of androgen deprivation in T-medium supplemented with 10% of charcoal-stripped serum. OCM and C4-2B CM were added to C4-2B FR cells seeded at a confluency of 10^4^ in 96-well plates for AR activity assay (see paragraph below) and 6 × 10^4^ in 24-well plates for cell cycle analysis (see paragraph below). For “direct co-cultures”, C4-2B FR cells were plated (10^4^ in 96 well plates) on an OB layer for AR activity assay (see paragraph below) and GFP+ C4-2B cells were seeded (5 × 10^4^ in 24-well plates) on an OB layer for proliferation analysis. To generate growth curves, GFP signal cells were measured at 24 h, 48 h, 96 h, and 120 h using Nikon NIS-Elements microscope imaging software. GFP-fluorescent signal at each time point was normalized to GFP-fluorescent signal at t0. As control, C4-2B FR cells were cultured on the C4-2B layer (for AR activity) and GFP+ C4-2B cells were cultured on the C4-2B layer (for proliferation analysis).

### Real-Time PCR

Total RNA was extracted by Trizol reagent (Invitrogen) according to the manufacturer’s instructions. cDNA was produced using the High-Capacity cDNA Reverse Transcription kit (Applied Biosystems) according to the manufacturer’s instructions. mRNA levels were measured by quantitative real-time polymerase chain reaction (qRT-PCR) using TaqMan Gene Expression Assays in the 7900HT Real-Time PCR System (Applied Biosystems). AR (Hs00171172_m1), KLK3 (Hs02576345_m1), TMPRSS2 (Hs05024838_m1), and MMP-1 (Hs00899658_m1) expression levels were normalized to the endogenous housekeeping gene Glucuronidase Beta (GUSβ) (Hs99999908_m1).

### Microarray

Gene expression profiling was performed using Clariom™ D Pico Assay, human (Affymetrix, USA) according to user guide. Quantile normalization and subsequent data processing were performed using Applied Biosystems™ Transcriptome Analysis Console (TAC) Software (Affymetrix, USA). A volcano plot representing differentially regulated genes was generated using R software (Vienna University of Economics and Business, Austria). According to the results of microarray, RNAs with fold change > 1.5 were marked as significantly differentially expressed genes (21). Gene set enrichment analysis was performed using WEBGESTALT (22).

### Western Blot

C4-2B FR cells were lysed in RIPA buffer (Sigma-Aldrich) with protease inhibitor cocktail (Sigma-Aldrich) and phosphatase inhibitor cocktail (Sigma-Aldrich). Protein concentration was measured using a DC protein assay (Bio-Rad) following the manufacturer’s instruction. AR primary antibody (Rabbit mAb, Cell Signaling), β-actin primary antibody (Mouse mAb, Sigma), and secondary HRP-conjugated anti-Rabbit or anti-Mouse IgG Ab (Cell Signaling) were used. Immunoreactive bands were visualized by ChemiDoc MTP Imaging System (Bio-Rad) and their intensity was quantified using ImageJ software.

### AR Luciferase Activity Assay

C4-2B FR cells were treated with OCM or C4-2B CM. After 24 h, AR activation was quantified using Dual-Luciferase Reporter Assay (Promega) following the manufacturer’s instructions. Firefly and Renilla luciferase signals were measured sequentially by a spectrofluorometer (Tecan Infinite M200Pro). AR activity was determined normalizing firefly luciferase signal with Renilla luciferase signal (constitutively expressed signal). Specificity of the luciferase signal was checked treating C4-2B FR cells with AR agonist R1881 (1 nM) and AR-antagonist enzalutamide (35 μM) (**Supplementary Figure 1**).

### Cell Cycle Analysis

**Figure 1:**
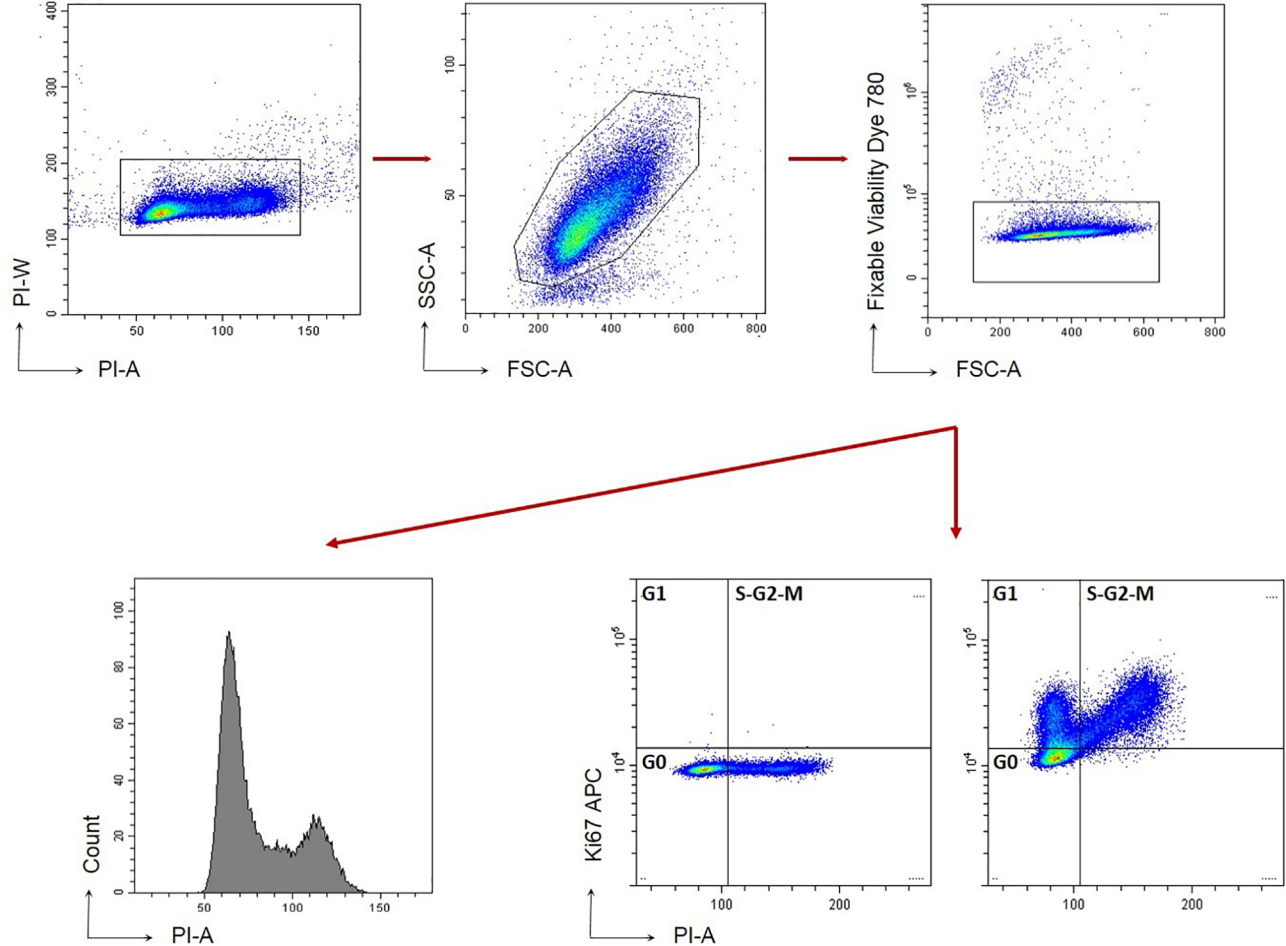
Gating strategy for C4-2B cell cycle. Cells are stained with FVD to exclude death cells (FVD+). Gating strategy based on Forward and Side scatter is shown.

Cell cycle analysis was performed on C4-2B FR cells treated with OCM or C4-2B CM for 96 h. Cell cycle was analyzed using the following gating strategy (**Figure 1**) (23). Briefly, cells were fixed and permeabilized with Foxp3/Transcription Factor Staining Buffer Set (Thermofisher eBioscience) for intracellular staining with anti-Ki67-APC antibody (clone 20Raj1 eBioscience) and a Propidium Iodide (PI) solution (50 μg/ml PI+ 40 ng/ml RNAseA+ 0.1% of Triton) (Sigma). Dead cell exclusion was performed with Fixable Viability Dye conjugated with eFluor780 fluorochrome (Affimetrix eBioscience). Samples were analyzed by CytoFlex instrument (Beckman Coulter) and using CytExpert Software, v.2.1. Raw data of cell cycle phases (percentage) are summarized in **Supplementary Figure 2**.

### Proteomic Assay

Proteomic profile analysis was performed on OCM or C4-2B CM. A panel of 507 human target proteins was analyzed using the human antibody Array Membrane Kit (RayBiotech) according to the manufacturer’s instructions. Band signal was detected by ChemiDoc MTP Imaging System (Bio-Rad), and their intensity was quantified using ImageLab Software (Bio-Rad).

### Statistical Analysis

Data were analyzed using the Student’s *t*-test and one-way ANOVA test followed by Tukey’s multiple comparison tests. The graphics processing and statistical tests were performed using the program GraphPad Prism (San Diego, CA).

## Results

### Osteoblasts Modulate CRPC Cell Gene Profile

**Figure 2:**
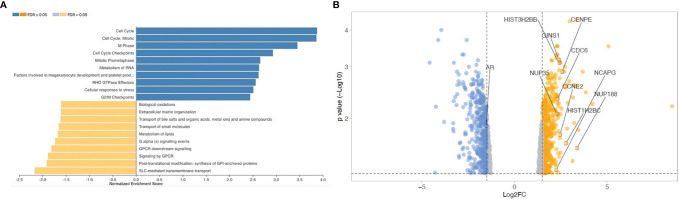
Transcriptomic analysis on C4-2B FR treated with OCM and C4-2B CM. **(A)** Gene set enrichment analysis and **(B)** Volcano plot of down- (blue) and upregulated genes (red) in C4-2B FR treated with OCM (fold change ˃1.5). Top 10 genes ranked by enrichment score for gene set “cell cycle R-HAS-1640170 REACTOME” and androgen receptor (AR) are labeled.

To evaluate if OB-secreted factors influence gene expression profile of CRPC cells, transcriptomic analysis was performed on C4-2B cells treated with OCM and C4-2B CM (as control). Pathway enrichment analysis revealed a significant upregulation of cell cycle signaling pathways [false discovery rate (FDR) adjusted *p*-value ≤ 0.05] in C4-2B cells treated with OCM compared to control, suggesting that OCM could promote cancer proliferation. Surprisingly, AR, which represents the major driver in prostate cancer proliferation, resulted among the genes that were significantly downregulated (**Figure 2**).

### Soluble Osteoblast Factors Inhibit AR and Promote CRPC Cell Proliferation

**Figure 3:**
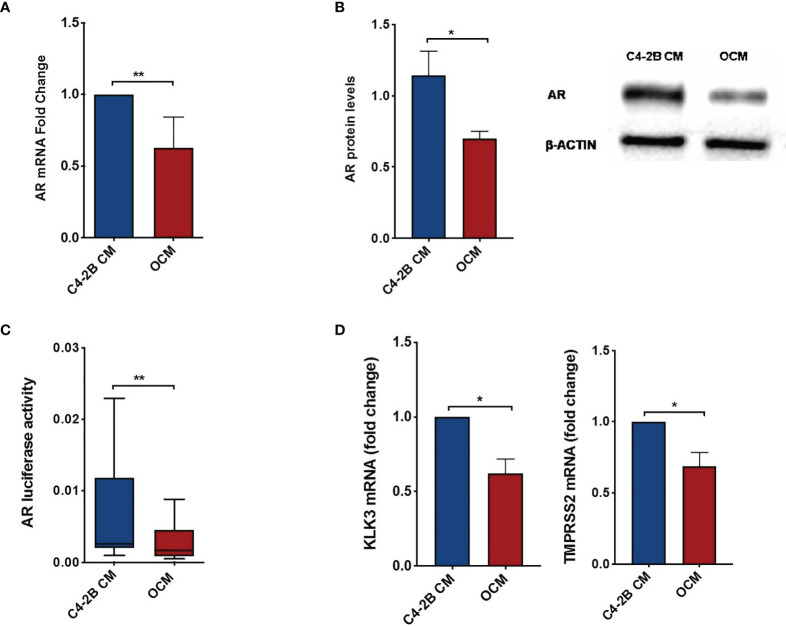
AR and AR-target gene expression in C4-2B FR cells after OCM treatment. **(A)** AR mRNA levels normalized for the housekeeping β-glucoronidase (GUS-β). Values are expressed as fold change relative to the control. **(B)** Representative image and schematic representation of AR protein expression normalized for the housekeeping β-actin. **(C)** AR activity of C4-2B FR cells measured as firefly luciferase signal normalized with Renilla luciferase signal (housekeeping control). **(D)** KLK3 and TMPRSS2 mRNA levels were normalized for the housekeeping β-glucoronidase (GUS-β). Values are expressed as fold change relative to the control.*p < 0.05; **p < 0.001.

**Figure 4:**
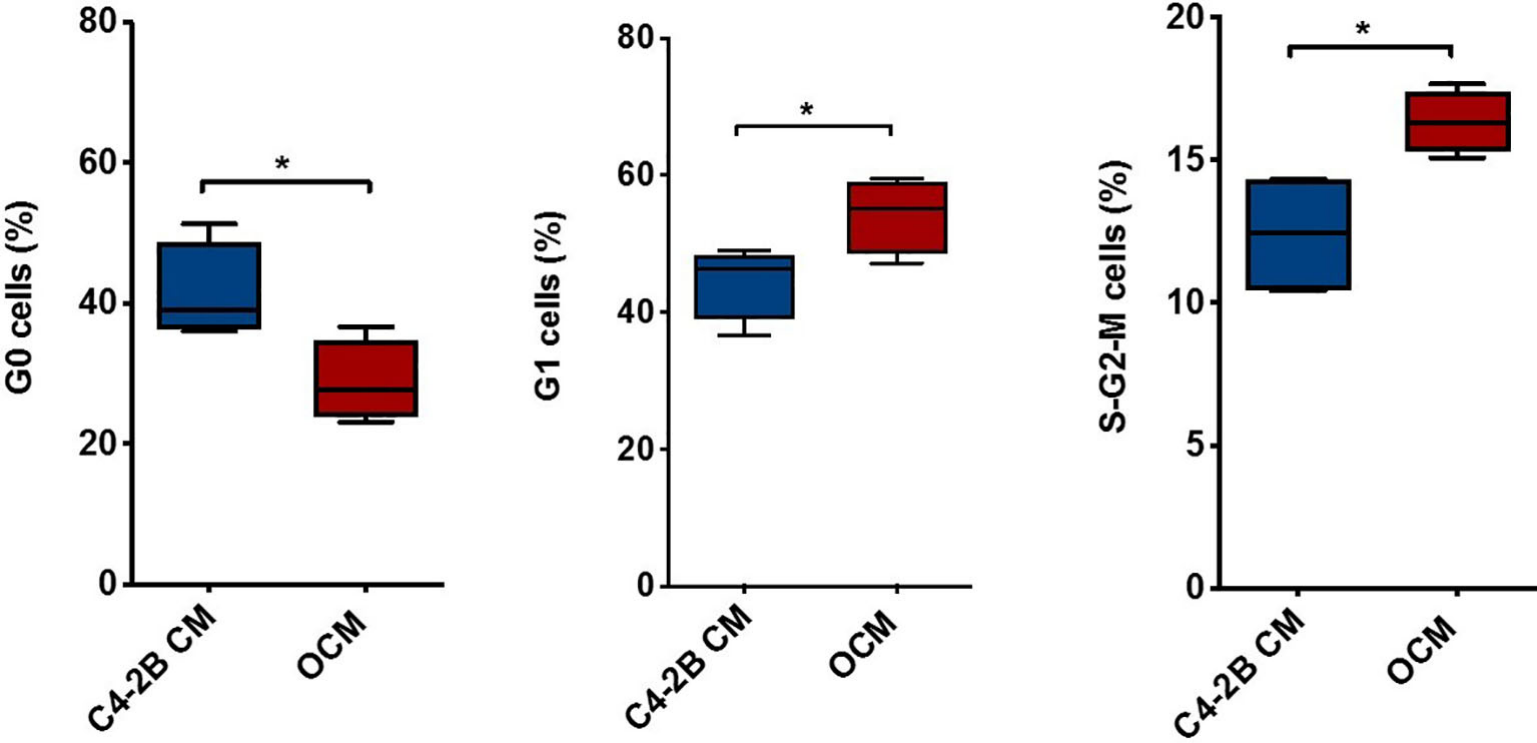
Combination of PI and Ki-67 staining in C4-2B FR cells treated with OCM. Schematic representation of C4-2B cells percentage in G0, G1, and S-G2-M phases after OCM treatment with average value (bar). Cytometry profiles are representative of data from 4 different experiments. *p < 0.05.

To dissect the effect of OB-secreted factors on AR signaling, we performed an “indirect” co-culture treating C4-2B FR cells with OCM or with C4-2B CM. Data confirmed that AR expression was downregulated in terms of mRNA (*p* = 0.0047), protein levels (*p* = 0.0247), and AR activity (*p* = 0.0029) when C4-2B FR cells grew in the presence of OCM compared to control (C4-2B CM) (**Figures 3A–C**). In addition, we found that OCM treatment significantly reduced mRNA levels of AR target genes such as Kallikrein Related Peptidase 3 (KLK3) (*p* = 0.02) and Transmembrane Serine Protease 2 (TMPRSS2) (*p* = 0.06) in C4-2B FR cells (**Figure 3D**). Next, we evaluated the effect of OCM on CRPC cell proliferation by flow cytometry. Data showed that OCM treatment increased the percentage of C4-2B in G1 (*p* = 0.0477) and S-G2-M (*p* = 0.0185) phases, but reduced the percentage of Ki-67-PI-resting cells in G0 phase (*p* = 0.0313) (**Figure 4**).

**Figure 5:**
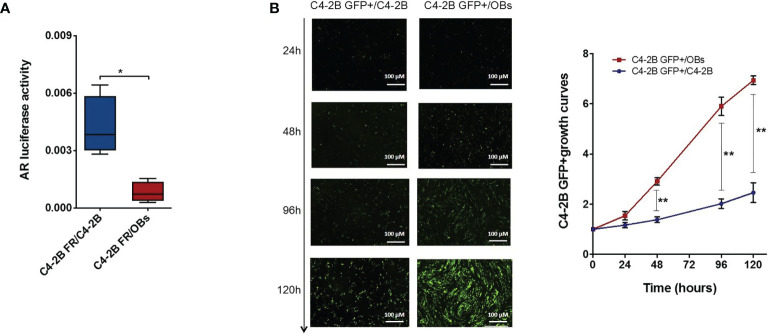
AR activity C4-2B FR cells and proliferation analysis of C4-2B GFP+ cells in “direct” co-culture with OBs. **(A)** AR activity measured as firefly luciferase signal normalized with Renilla luciferase signal (housekeeping control). **(B)** Representative images of direct co-cultures captured at 24, 48, 96, and 120 h after C4-2B GFP+ cell (green) seeding; scale bar = 100 μm. Proliferation curves C4-2B GFP+ cells co-cultured with OBs (red) or C4-2B as control (blue). GFP+ signal at each time point was normalized with GFP signal at baseline *p < 0.05; **p < 0.001.

We obtained similar results co-culturing CRPC cells and OBs in cell–cell contact models. In particular, we set up a “direct” OBs/C4-2B FR co-culture seeding C4-2B FR cells on an OB monolayer or C4-2B FR cells on a C4-2B cell monolayer as control. Data confirmed a significant reduction of AR activity (*p* = 0.020) (**Figure 5A**) and a concomitant increase of C4-2B GFP+ cell proliferation when cancer cells grew in the presence of OBs. In particular, the presence of OBs augmented C4-2B GFP+ growth at 48 h (*p* = 0.006), 96 h (*p* = 0.006), and 120 h (*p* = 0.004) (**Figure 5B**).

### Analysis of Osteoblast Secretome Identifies MMP-1 as a Key Mediator of CRPC Proliferation AR Independent

**Figure 6:**
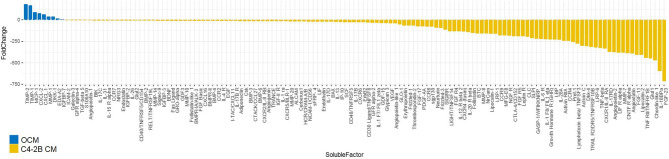
Waterfall plot of soluble molecules differentially expressed in OCM compared to C4-2B CM (expressed as fold change). Proteomic analysis was performed on OCM pooled from three different donors and C4-2B CM from three different cell passages; molecules overexpressed in OCM with a fold change > 2 (blue) and downregulated fold change < 2 (yellow) are reported.

To identify potential OB soluble factors involved in AR reduction and/or CRPC proliferation, an extensive proteomic analysis was performed on OCM and C4-2B CM. Secretome analysis identified a subset of 154 molecules that were differentially expressed between OCM and C4-2B CM. After filtering analysis based on the factors overexpressed in OCM, with a fold change > 2, we identified 9 soluble molecules: TIMP metallopeptidase inhibitor 2 (TIMP-2), TIMP metallopeptidase inhibitor 1 (TIMP-1), Monocyte chemoattractant protein-1 (MCP-1), C-X-C Motif Chemokine Ligand 2 (CXCL-2), C-X-C Motif Chemokine Ligand 1 (CXCL-1), Matrix metalloproteinase-1 (MMP-1), Dickkopf-1 (DKK-1), Ectodysplasin A2 (EDA-A2), and Insulin Like Growth Factor Binding Protein 7 (IGFBP-7) (**Figure 6**).

**Figure 7:**
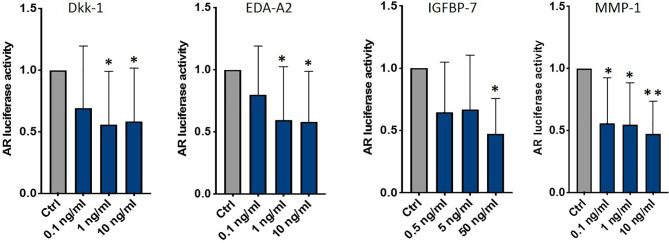
AR activity of C4-2B FR cultured in C4-2B CM supplemented with DKK-1, EDA-A2, IGFBP-7, and MMP-1; *p < 0.05; **p < 0.001.

To investigate if one or some of these factors influenced AR activity, we treated C4-2B FR cells with C4-2B CM adding different dosages of each molecule (**Supplementary Figure 3A**). We found that DKK-1 (1 ng/ml *p* = 0.03; 10 ng/ml *p* = 0.04), EDA-A2 (1 ng/ml *p* = 0.04; 10 ng/ml *p* = 0.03), IGFBP-7 (50 ng/ml *p* = 0.03), and MMP-1 (0.1 ng/ml *p* = 0.006; 1 ng/ml *p* = 0.002; 10 ng/ml *p* = 0.0001) significantly reduced AR activity (**Figure 7**).

**Figure 8:**
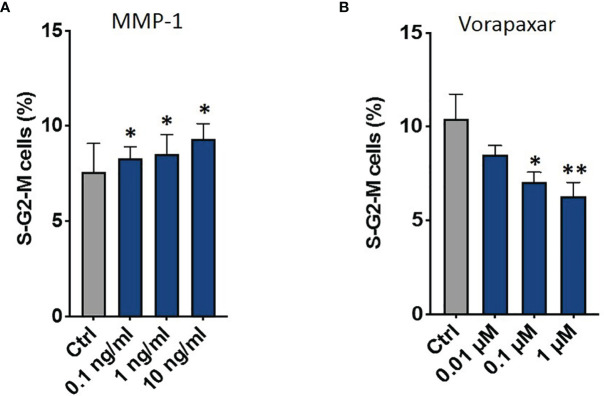
Combination of PI and Ki-67 staining in C4-2B FR cells treated with OCM. Schematic representation of C4-2B cells percentage in S-G2-M phases treated with C4-2B CM supplemented with MMP-1 **(A)** and with OCM in the presence of different dosages of vorapaxar **(B)**. *p < 0.05; **p < 0.001.

Then, we tested the effect of these factors on CRPC proliferation through flow cytometry. Data showed that only MMP-1 was able to increase S-G2-M phases (1 ng/ml *p* = 0.009; 10 ng/ml *p* = 0.033) (**Figure 8A** and **Supplementary Figure 3B**). Based on these results, we performed further analyses to confirm the direct involvement of MMP-1 in AR signal downregulation and CRPC proliferation. In particular, data showed that MMP-1 significantly reduced AR mRNA levels (0.1 ng/ml *p* = 0.024; 1 ng/ml *p* < 0.001; 10 ng/ml *p* < 0.001) (**Supplementary Figure 4**). Moreover, to confirm that MMP-1 is directly involved in CRPC proliferation through the activation of its receptor, Protease-activated receptor-1 (PAR-1), we treated C4-2B FR with OCM plus Vorapaxar (0.01 μM, 0.1 μM, and 1 μM), a specific PAR-1 inhibitor. As shown in **Figure 8B**, Vorapaxar significantly reduced S-G2-M cell cycle phases (0.1 μM *p* = 0.014; 1 μM *p* = 0.0087), confirming that MMP-1/PAR-1 could be one of the signaling pathways involved in AR-independent CRPC proliferation.

## Discussion

Our results provide a novel AR-independent mechanism of CRPC proliferation mediated by OBs. In the current study, we demonstrated that OB-secreted factors reduced AR activity, but, surprisingly, induced the growth of prostate cancer cells. Proteomic analysis identified some soluble factors highly expressed in OCM compared to C4-2B CM, and, among these, four molecules were able to reduce AR activation, namely, DKK-1, MMP-1, EDA-A2, and IGFBP-7. Intriguingly, MMP-1 had the ability to decrease AR signal and, concomitantly, enhance prostate cancer cell proliferation. However, the increase in C4-2B cell growth induced by MMP-1 did not reach the proliferation levels observed after OCM treatment, suggesting that MMP-1 may not be the only factor responsible for AR-independent CRPC proliferation. MMP-1 is a potent agonist for PAR1, a G-protein-coupled receptor (GPCR) that plays critical roles in thrombosis, inflammation, and vascular biology (24). Moreover, several lines of evidence reported that PAR1 is also involved in the invasive and metastatic processes of various cancers (25–29). Thus, to evaluate if the MMP-1/PAR-1 axis was involved in OB-mediated CRPC proliferation, we treated C4-2B cells with Vorapaxar, a specific PAR-1 inhibitor. Intriguingly, C4-2B cell cycle was significantly reduced after treatment, confirming the potential role of MMP-1/PAR-1 pathway in tumor growth. Some studies reported that MMP-1 was mainly secreted by stromal cells, such as OBs and fibroblasts, in prostate and breast tumor microenvironment and promoted cancer cell migration and invasion through PAR-1 signaling (30, 31). In addition, cDNA microarray analysis revealed an increased expression of PAR1 on bone-derived prostate cancer cell lines (32), confirming the role of bone microenvironment in promoting MMP-1/PAR1 pathway activation.

Similar to the current study, previous papers reported that OBs contribute to prostate tumor proliferation in *in vitro* co-culture models of OBs and prostate cancer cell lines (33–36). In particular, Blaszczyket et al. demonstrated that interleukin-6 secreted by OBs stimulated the androgen-independent proliferation of prostate cancer cells by a mechanism that was partially AR dependent (33). However, data were obtained on LNCaP cells, a hormone-sensitive prostate cancer cell line that differs from C4-2B cells, which represent the best characterized castration-resistant bone metastatic model (37). In addition, Thulin and colleagues reported a key role of OBs as mediators of CRPC cell growth in bone through the stimulation of the intratumoral steroidogenesis. Differently from us, the authors used immortalized murine calvarial cell line MC3T3-E1 that did not completely resemble the physiological bone microenvironment (34). More recently, another study proposed an interesting *in vitro* 3D model to investigate the OBs/prostate cancer cell crosstalk. This more complex co-culture system highlighted the crucial involvement of OBs in prostate cancer progression (35). Moreover, preclinical studies showed that prostate cancer cells induced a strong osteogenic response (36) as well as the co-injection of bone stromal cells and human prostate cancer cells enhanced tumor formation in mice (17, 38). However, how osteoblasts supported tumor growth was not fully clarified. Sung et al. (17) identified extracellular matrix proteins versican and tenascin and chemokine ligands CXCL5 and CXCL16, released by stromal cells, as potential mediators of prostate cancer cell proliferation. Moreover, transcriptomic analysis of the stroma compartment of bones xenografted with human CRPC cells showed that some genes were upregulated in mouse stromal cells including PTN, EPHA3, and FSCN1 (39). These data suggest that a combination of stromal secreted factors, rather than one, could provide a support for prostate cancer cells. Taken together, these lines of evidence highlight the crucial role of bone microenvironment in supporting prostate cancer progression, but the identification of the cellular and molecular determinants remains an important unsolved question.

In this complex but fascinating scenario, our results reveal a novel potential mechanism of proliferation induced by OBs through an AR-independent mechanisms. Paradoxically, we found that AR signaling remained very low in bone microenvironment, and these findings have been confirmed at different levels, i.e., mRNA, protein, and functional activity. Although we consider this phenomenon real, we are not able to insert it in any biological frameworks given by previous literature data. Indeed, to our knowledge, no identified pathway can simultaneously inhibit AR and stimulate CRPC proliferation. Thus, MMP-1/PAR-1 could be one of the potential pathways able to promote AR-independent CRPC proliferation. However, further extensive investigations are warranted to completely elucidate the mechanisms that fully explain the data we observed. This represents the major limitation of the study.

To date, all mechanisms identified as responsible for CRPC progression or resistance to antiandrogens directly involved AR signaling. These include AR amplification and hypersensitivity (40), AR mutations (41), alterations in coactivators/corepressors (42), androgen-independent AR activation (43), intratumoral and alternative androgen production (44), and AR splice variants (45, 46). Here, we found that the AR axis could not be the only driver of disease progression, in bone metastatic niche, but it could be bypassed by alternative signaling pathways. Indeed, this inhibitory effect exerted by OB on AR activity could induce tumor cells to promote alternative molecular pathways of proliferation. Thus, bone microenvironment could provide novel potential mechanisms of resistance to second-generation AR inhibitors and, in particular, the MMP-1/PAR-1 axis could represent a new druggable pathway deserving of further studies.

## Data Availability Statement

The original contributions presented in the study are included in the article/**Supplementary Material**. Further inquiries can be directed to the corresponding author.

## Author Contributions

GR: Methodology and Data analysis. SS: Methodology and Data analysis. MI: Data analysis and Writing—Original draft preparation. ER: Methodology. BV: Data analysis. GT: Supervision and Reviewing. FP: Study design and Data interpretation. DS: Critical revision of the manuscript. All authors contributed to the article and approved the submitted version.

## Conflict of Interest

The authors declare that the research was conducted in the absence of any commercial or financial relationships that could be construed as a potential conflict of interest.

## Publisher’s Note

All claims expressed in this article are solely those of the authors and do not necessarily represent those of their affiliated organizations, or those of the publisher, the editors and the reviewers. Any product that may be evaluated in this article, or claim that may be made by its manufacturer, is not guaranteed or endorsed by the publisher.

## Supplementary Material

Supplementary Figure 1 AR activity of C4-2B FR cells after treatment with R1881 (1nM) and enzalutamide (35μM) measured as firefly-luciferase signal normalized with renilla-luciferase signal. *p < 0.05.

Supplementary Figure 2 Percentage of cell cycle phases (raw data).

Supplementary Figure 3
**(A)** AR activity of C4-2B FR cultured in C4-2B CM supplemented with CXCL-1, CXCL-2, MCP-1, TIMP-1 and TIMP-2. **(B)** Schematic representation of C4-2B cells percentage in S-G2-M phases treated with C4-2B CM supplemented with DKK-1, IGFBP-7 and EDA-A2.

Supplementary Figure 4 MMP-1 mRNA levels normalized for the housekeeping β-glucoronidase (GUS-β). Values are expressed as fold change relative to the control. *p < 0.05; **p < 0.001.

## References

[1] Harris WP Mostaghel EA Nelson PS Montgomery B. Androgen Deprivation Therapy: Progress in Understanding Mechanisms of Resistance and Optimizing Androgen Depletion. Nat Clin Pract Urol (2009) 6:76–85. doi: 10.1038/ncpuro1296 19198621 PMC2981403

[2] Chandrasekar T Yang JC Gao AC Evans CP. Mechanisms of Resistance in Castration-Resistant Prostate Cancer (CRPC). Trans Androl Urol (2015) 4:365–80. doi: 10.3978/j.issn.2223-4683.2015.05.02 PMC4708226 26814148

[3] Rice MA Malhotra SV Stoyanova T. Second-Generation Antiandrogens: From Discovery to Standard of Care in Castration Resistant Prostate Cancer. Front Oncol (2019) 28:801. doi: 10.3389/fonc.2019.00801 PMC6723105 31555580

[4] Wadosky KM Koochekpour S. Androgen Receptor Splice Variants and Prostate Cancer: From Bench to Bedside. Oncotarget (2017) 8:18550–76. doi: 10.18632/oncotarget.14537 PMC5392349 28077788

[5] Locke JA Guns ES Lubik AA Adomat HH Hendy SC Wood CA. Androgen Levels Increase by Intratumoral *De Novo* Steroidogenesis During Progression of Castration-Resistant Prostate Cancer. Cancer Res (2008) 68:6407–15. doi: 10.1158/0008-5472.CAN-07-5997 18676866

[6] Takayama KI. Splicing Factors Have an Essential Role in Prostate Cancer Progression and Androgen Receptor Signaling. Biomolecules (2019) 9:131. doi: 10.3390/biom9040131 PMC6523118 30939845

[7] Kobayashi T Inoue T Kamba T Ogawa O. Experimental Evidence of Persistent Androgen-Receptor-Dependency in Castration-Resistant Prostate Cancer. Int J Mol Sci (2013) 14:15615–35. doi: 10.3390/ijms140815615 PMC3759876 23896594

[8] Hensel J Thalmann GN. Biology of Bone Metastases in Prostate Cancer. Urology (2016) 92:6–13. doi: 10.1016/j.urology.2015.12.039 26768714

[9] Coleman RE. Clinical Features of Metastatic Bone Disease and Risk of Skeletal Morbidity. Clin Cancer Res (2006) 12:6243s–9s. doi: 10.1158/1078-0432.CCR-06-0931 17062708

[10] Mundy GR. Metastasis to Bone : Causes, Consequences and Therapeutic Opportunities. Nat Rev Cancer (2002) 2:584–93. doi: 10.1038/nrc867 12154351

[11] Logothetis CJ Lin SH. Osteoblasts in Prostate Cancer Metastasis to Bone. Nat Rev Cancer (2005) 5:21–8. doi: 10.1038/nrc1528 15630412

[12] Turner CJ Edwards CM. The Role of the Microenvironment in Prostate Cancer-Associated Bone Disease. Curr Osteoporos Rep (2016) 14:170–7. doi: 10.1007/s11914-016-0323-2 27566487

[13] Mundy GR. Malignancy and the Skeleton. Horm Metab Res (1997) 29:120–7. doi: 10.1055/s-2007-979004 9137982

[14] Ottewell PD. The Role of Osteoblasts in Bone Metastasis. J Bone Oncol (2016) 5:124–7. doi: 10.1016/j.jbo.2016.03.007 PMC5063217 27761372

[15] Zhang S Wang J Bilen MA Lin SH Stupp SI Satcher RL. Modulation of Prostate Cancer Cell Gene Expression by Cell-to-Cell Contact With Bone Marrow Stromal Cells or Osteoblasts. Clin Exp Metastasis (2009) 26:993–1004. doi: 10.1007/s10585-009-9289-0 19787436

[16] Li Y Sikes RA Malaeb BS Yeung F Law A Graham SE. Osteoblasts can Stimulate Prostate Cancer Growth and Transcriptionally Down-Regulate PSA Expression in Cell Line Models. Urol Oncol (2011) 29:802–8. doi: 10.1016/j.urolonc.2009.09.016 20451417

[17] Sung SY Hsieh CL Law A Zhau HE Pathak S Multani AS. Coevolution of Prostate Cancer and Bone Stroma in Three-Dimensional Coculture: Implications for Cancer Growth and Metastasis. Cancer Res (2008) 68:9996–10003. doi: 10.1158/0008-5472.CAN-08-2492 19047182 PMC3105756

[18] Thalmann N Anezinis PE Chang SM Zhau HE Kim EE Hopwood VL. Androgen-Independent Cancer Progression and Bone Metastasis in the LNCaP Model of Human Prostate Cancer. Cancer Res (1994) 54:2577–82. 8168083

[19] Iuliani M Simonetti S Pantano F Ribelli G Di Martino A Denaro V. Antitumor Effect of Cabozantinib in Bone Metastatic Models of Renal Cell Carcinoma. Biology (Basel) (2021) 16:781. doi: 10.3390/biology10080781 PMC8389553 34440012

[20] Iuliani M Pantano F Buttigliero C Fioramonti M Bertaglia V Vincenzi B. Biological and Clinical Effects of Abiraterone on Anti-Resorptive and Anabolic Activity in Bone Microenvironment. Oncotarget (2015) 6:12520–8. doi: 10.18632/oncotarget.3724 PMC4494955 25904051

[21] Thomas JG Olson JM Tapscott SJ Zhao LP. An Efficient and Robust Statistical Modeling Approach to Discover Differentially Expressed Genes Using Genomic Expression Profiles. Genome Res (2001) 11:1227–36. doi: 10.1101/gr.165101 PMC311075 11435405

[22] Liao Y Wang J Jaehnig EJ Shi Z Zhang B. WebGestalt 2019: Gene Set Analysis Toolkit With Revamped UIs and APIs. Nucleic Acids Res (2019) 47:W199–205. doi: 10.1093/nar/gkz401 PMC6602449 31114916

[23] Simonetti S Natalini A Folgori A Capone S Nicosia A Santoni A. Antigen-Specific CD8 T Cells in Cell Cycle Circulate in the Blood After Vaccination. Scand J Immunol (2019) 89:e12735. doi: 10.1111/sji.12735 30488973 PMC6850756

[24] Gieseler F Ungefroren H Settmacher U Hollenberg MD Kaufmann R. Proteinase-Activated Receptors (PARs) - Focus on Receptor-Receptor-Interactions and Their Physiological and Pathophysiological Impact. Cell Commun Signal (2013) 11:86. doi: 10.1186/1478-811X-11-86 24215724 PMC3842752

[25] Even-Ram S Uziely B Cohen P Grisaru-Granovsky S Maoz M Ginzburg Y. Thrombin Receptor Overexpression in Malignant and Physiological Invasion Processes. Nat Med (1998) 4:909–14. doi: 10.1038/nm0898-909 9701242

[26] Yang E Cisowski J Nguyen N O'Callaghan K Xu J Agarwal A. Dysregulated Protease Activated Receptor 1 (PAR1) Promotes Metastatic Phenotype in Breast Cancer Through HMGA2. Oncogene (2016) 35:1529–40. doi: 10.1038/onc.2015.217 PMC6818098 26165842

[27] Kancharla A Maoz M Jaber M Agranovich D Peretz T Grisaru-Granovsky S. PH Motifs in PAR1&2 Endow Breast Cancer Growth. Nat Commun (2015) 6:8853. doi: 10.1038/ncomms9853 26600192 PMC4673491

[28] Salah Z Maoz M Pokroy E Lotem M Bar-Shavit R Uziely B. Protease-Activated Receptor-1 (Hpar1), A Survival Factor Eliciting Tumor Progression. Mol Cancer Res (2007) 5:229–40. doi: 10.1158/1541-7786.MCR-06-0261 17374729

[29] Zhang Y Zhan H Xu W Yuan Z Lu P Zhan L. Upregulation of Matrix Metalloproteinase-1 and Proteinase-Activated Receptor-1 Promotes the Progression of Human Gliomas. Pathol Res Pract (2011) 207:24–9. doi: 10.1016/j.prp.2010.10.003 21087829

[30] Boire A Covic L Agarwal A Jacques S Sherifi S Kuliopulos A. PAR1 is a Matrix Metalloprotease-1 Receptor That Promotes Invasion and Tumorigenesis of Breast Cancer Cells. Cell (2005) 120:303–13. doi: 10.1016/j.cell.2004.12.018 15707890

[31] Forbes K Webb MA Sehgal I. Growth Factor Regulation of Secreted Matrix Metalloproteinase and Plasminogen Activators in Prostate Cancer Cells, Normal Prostate Fibroblasts and Normal Osteoblasts. Prostate Cancer Prostatic Dis (2003) 6:148–53. doi: 10.1038/sj.pcan.4500640 12806374

[32] Chay CH Cooper CR Gendernalik JD Dhanasekaran SM Chinnaiyan AM Rubin MA. A Functional Thrombin Receptor (PAR1) Is Expressed on Bone-Derived Prostate Cancer Cell Lines. Urology (2002) 60:760–5. doi: 10.1016/S0090-4295(02)01969-6 12429291

[33] Blaszczyk N Masri BA Mawji NR Ueda T McAlinden G Duncan CP. Osteoblast-Derived Factors Induce Androgen-Independent Proliferation and Expression of Prostate-Specific Antigen in Human Prostate Cancer Cells. Clin Cancer Res (2004) 10:1860–9. doi: 10.1158/1078-0432.CCR-0974-3 15014041

[34] Hagberg Thulin M Nilsson ME Thulin P Céraline J Ohlsson C Damber JE. Osteoblasts Promote Castration-Resistant Prostate Cancer by Altering Intratumoral Steroidogenesis. Mol Cell Endocrinol (2016) 422:182–91. doi: 10.1016/j.mce.2015.11.013 26586211

[35] Bock N Shokoohmand A Kryza T Röhl J Meijer J Tran PA. Engineering Osteoblastic Metastases to Delineate the Adaptive Response of Androgen-Deprived Prostate Cancer in the Bone Metastatic Microenvironment. Bone Res (2019) 7:13. doi: 10.1038/s41413-019-0049-8 31044095 PMC6486620

[36] Li ZG Mathew P Yang J Starbuck MW Zurita AJ Liu J. Androgen Receptor-Negative Human Prostate Cancer Cells Induce Osteogenesis in Mice Through FGF9-Mediated Mechanisms. J Clin Invest (2008) 118:2697–710. doi: 10.1172/JCI33093 PMC2447924 18618013

[37] Pfitzenmaier J Quinn JE Odman AM Zhang J Keller ET Vessella RL. Characterization of C4-2 Prostate Cancer Bone Metastases and Their Response to Castration. J Bone Miner Res (2003) 18:1882–8. doi: 10.1359/jbmr.2003.18.10.1882 14584899

[38] Gleave M Hsieh JT Gao CA von Eschenbach AC Chung LW. Acceleration of Human Prostate Cancer Growth *In Vivo* by Factors Produced by Prostate and Bone Fibroblasts. Cancer Res (1991) 51:3753–61. 1712249

[39] Ozdemir BC Hensel J Secondini C Wetterwald A Schwaninger R Fleischmann A. The Molecular Signature of the Stroma Response in Prostate Cancer-Induced Osteoblastic Bone Metastasis Highlights Expansion of Hematopoietic and Prostate Epithelial Stem Cell Niches. PloS One (2014) 9:el 14530. doi: 10.1371/journal.pone.0114530 PMC4259356 25485970

[40] Gregory CW Johnson RT Jr Mohler JL French FS Wilson EM. Androgen Receptor Stabilization in Recurrent Prostate Cancer Is Associated With Hypersensitivity to Low Androgen. Cancer Res (2001) 61:2892–8. 11306464

[41] Taylor BS Schultz N Hieronymus H Taylor BS Schultz N Hieronymus H. Integrative Genomic Profiling of Human Prostate Cancer. Cancer Cell (2010) 18:11–22. doi: 10.1016/j.ccr.2010.05.026 20579941 PMC3198787

[42] Wolf IM Heitzer MD Grubisha M DeFranco DB. Coactivators and Nuclear Receptor Transactivation. J Cell Biochem (2008) 104:1580–6. doi: 10.1002/jcb.21755 18393355

[43] Wang Q Li W Zhang Y Yuan X Xu K Yu J. Androgen Receptor Regulates a Distinct Transcription Program in Androgen-Independent Prostate Cancer. Cell (2009) 138:245–56. doi: 10.1016/j.cell.2009.04.056 PMC2726827 19632176

[44] Montgomery RB Mostaghel EA Vessella R Hess DL Kalhorn TF Higano CS. Maintenance of Intratumoral Androgens in Metastatic Prostate Cancer: A Mechanism for Castration-Resistant Tumor Growth. Cancer Res (2008) 68:4447–54. doi: 10.1158/0008-5472.CAN-08-0249 PMC2536685 18519708

[45] Guo Z Yang X Sun F Jiang R Linn DE Chen H. A Novel Androgen Receptor Splice Variant Is Up-Regulated During Prostate Cancer Progression and Promotes Androgen Depletion-Resistant Growth. Cancer Res (2009) 69:2305–13. doi: 10.1158/0008-5472.CAN-08-3795 PMC2672822 19244107

[46] Obinata D Lawrence MG Takayama K Choo N Risbridger GP Takahashi S. Recent Discoveries in the Androgen Receptor Pathway in Castration-Resistant Prostate Cancer. Front Oncol (2020) 8:581515. doi: 10.3389/fonc.2020.581515 PMC7578370 33134178

