# Supplementary figures and images for: Osteoblasts Promote Prostate Cancer Cell Proliferation Through Androgen Receptor Independent Mechanisms
Source: Front Oncol. 2021 Dec 13;11:789885. doi: 10.3389/fonc.2021.789885 (PMC8711264; doi:10.3389/fonc.2021.789885)

# AR luciferase activity

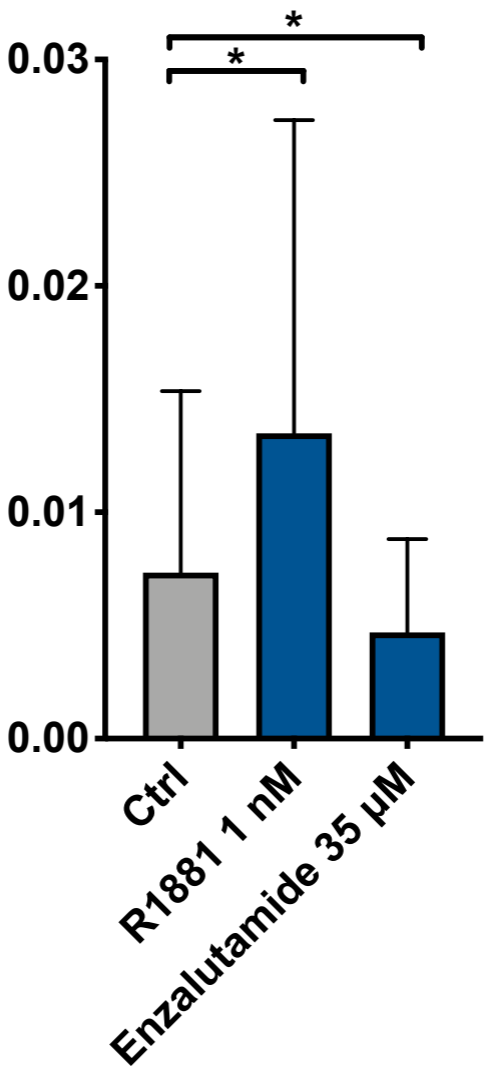

Supplement: Supplementary Figure 1 — AR activity of C4-2B FR cells after treatment with R1881 (1nM) and enzalutamide (35μM) measured as firefly-luciferase signal normalized with renilla-luciferase signal. *p < 0.05. [file DataSheet_1.pdf]

**A**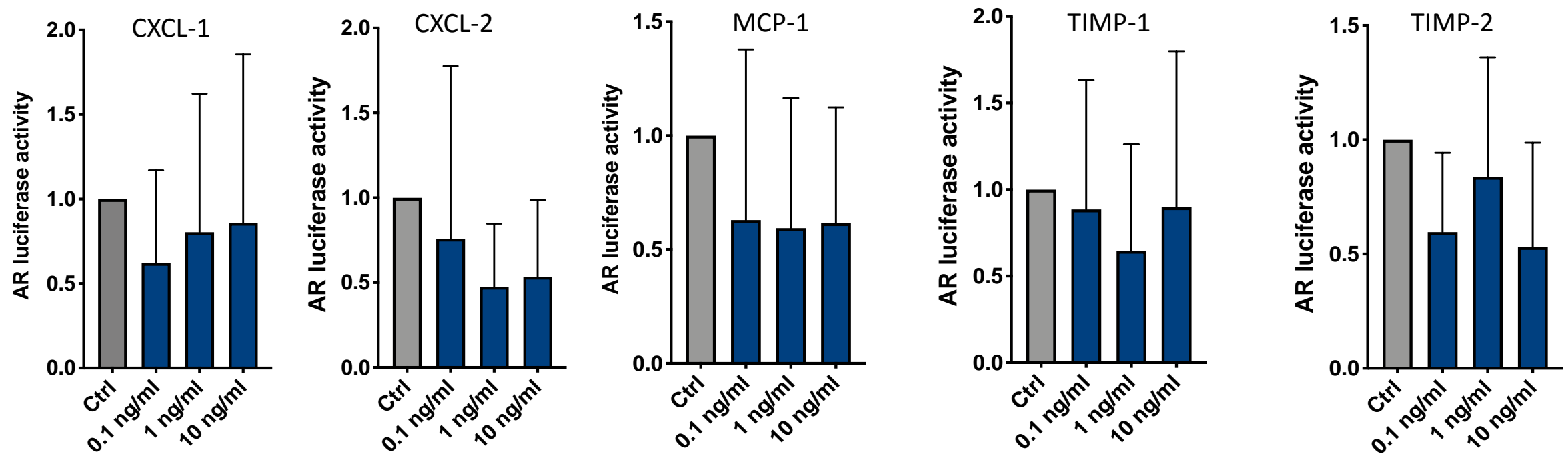**B**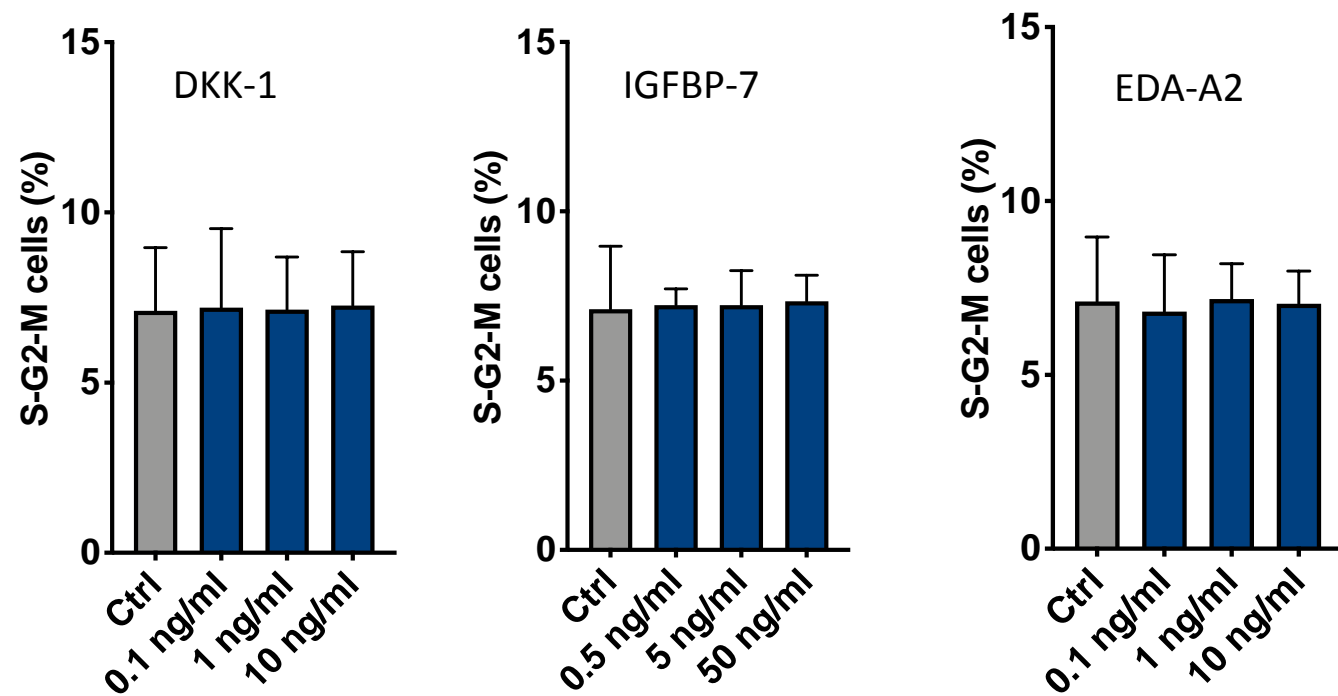

Supplement: Supplementary Figure 3 — (A) AR activity of C4-2B FR cultured in C4-2B CM supplemented with CXCL-1, CXCL-2, MCP-1, TIMP-1 and TIMP-2. (B) Schematic representation of C4-2B cells percentage in S-G2-M phases treated with C4-2B CM supplemented with DKK-1, IGFBP-7 and EDA-A2. [file DataSheet_3.pdf]

mRNA (fold change)

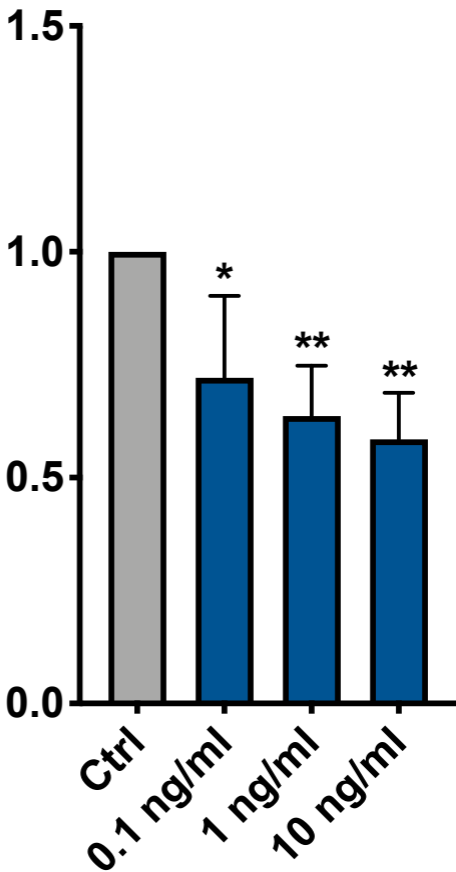

Supplement: Supplementary Figure 4 — MMP-1 mRNA levels normalized for the housekeeping β-glucoronidase (GUS-β). Values are expressed as fold change relative to the control. *p < 0.05; **p < 0.001. [file DataSheet_4.pdf]
