# Supplementary material for: Osteoblasts Promote Prostate Cancer Cell Proliferation Through Androgen Receptor Independent Mechanisms
Source: Front Oncol. 2021 Dec 13;11:789885. doi: 10.3389/fonc.2021.789885 (PMC8711264; doi:10.3389/fonc.2021.789885)
Supplement: Supplementary Figure 2 — Percentage of cell cycle phases (raw data). [file DataSheet_2.pdf]

Raw data G0 cells (Figure 4)				
	Exp 1	Exp 2	Exp 3	Exp 4
C4-2B CM	38.8	37.7	51	
OCM	26.6	34.6	18.4	29.4

Raw data G1 cells (Figure 4)				
	Exp 1	Exp 2	Exp 3	Exp 4
C4-2B CM	48.8	48.1	36.4	
OCM	58.3	50.6	64	54.4

Raw data S-G2-M cells (Figure 4)				
	Exp 1	Exp 2	Exp 3	Exp 4
C4-2B CM	11.3	13.2	11.1	
OCM	15.3	16.7	17.2	17.3

Raw data S-G2-M cells (Figure 8)						
	Exp 1	Exp 2	Exp 3	Exp 4	Exp 5	Exp 6
Ctrl	7.11	6.15	8.78	7.56	7.8	8.13
MMP-1 0.1 ng/ml	8.2	7.63	9.15	7.8	7.85	9.35
MMP-1 1 ng/ml	8.5	7.83	9.87	8.1	8.47	9.47
MMP-1 10 ng/ml	10.03	9.08	9.87	8.25	8.49	10.15

Raw data S-G2-M cells (Figure 8)			
	Exp 1	Exp 2	Exp 3
Ctrl	9.1	10.5	11.7
Vorapaxar 0.01 μM	8	9	8.5
Vorapaxar 0.1 μM	7.6	6.6	7
Vorapaxar 1 μM	7.14	6	5.8

Raw data S-G_2-M cells (Supplementary Figure 3)							
	Exp 1	Exp 2	Exp 3	Exp 4	Exp 5	Exp 6	Exp 7
Ctrl	8.11	5.15	4.25	8.78	6.56	7.8	9.13
DKK-1 0.1 ng/ml	8.3	4.53	8.77				
DKK-1 1 ng/ml	7.59	5.43	8.42				
DKK-1 10 ng/ml	7.83	5.49	8.49				
IGFBP-7 0.5 ng/ml	6.89	7.57	7.36				
IGFBP-7 5 ng/ml	7.95	6.52	6.98				
IGFBP-7 50 ng/ml	6.8	7.89	8.1				
EDA-A2 0.1 ng/ml	6.94	5.14	8.39				
EDA-A2 1 ng/ml	7.05	6.23	8.26				
EDA-A2 10 ng/ml	7.71	5.98	7.46				
